# Phosphorylation and Transport in the Na-K-2Cl Cotransporters, NKCC1 and NKCC2A, Compared in HEK-293 Cells

**DOI:** 10.1371/journal.pone.0017992

**Published:** 2011-03-25

**Authors:** Anke Hannemann, Peter W. Flatman

**Affiliations:** Centre for Integrative Physiology, College of Medicine and Veterinary Medicine, The University of Edinburgh, Edinburgh, United Kingdom; Institut National de la Santé et de la Recherche Médicale, France

## Abstract

Na-K-2Cl cotransporters help determine cell composition and volume. NKCC1 is widely distributed whilst NKCC2 is only found in the kidney where it plays a vital role reabsorbing 20% of filtered NaCl. NKCC2 regulation is poorly understood because of its restricted distribution and difficulties with its expression in mammalian cell cultures. Here we compare phosphorylation of the N-termini of the cotransporters, measured with phospho-specific antibodies, with bumetanide-sensitive transport of K^+^ (^86^Rb^+^) (activity) in HEK-293 cells stably expressing fNKCC1 or fNKCC2A which were cloned from ferret kidney. Activities of transfected transporters were distinguished from those of endogenous ones by working at 37°C. fNKCC1 and fNKCC2A activities were highest after pre-incubation of cells in hypotonic low-[Cl^−^] media to reduce cell [Cl^−^] and volume during flux measurement. Phosphorylation of both transporters more than doubled. Pre-incubation with ouabain also strongly stimulated fNKCC1 and fNKCC2A and substantially increased phosphorylation, whereas pre-incubation in Na^+^-free media maximally stimulated fNKCC1 and doubled its phosphorylation, but inhibited fNKCC2A, with a small increase in its phosphorylation. Kinase inhibitors halved phosphorylation and activity of both transporters whereas inhibition of phosphatases with calyculin A strongly increased phosphorylation of both transporters but only slightly stimulated fNKCC1 and inhibited fNCCC2A. Thus kinase inhibition reduced phosphorylation and transport, and transport stimulation was only seen when phosphorylation increased, but transport did not always increase with phosphorylation. This suggests phosphorylation of the N-termini determines the transporters' potential capacity to move ions, but final activity also depends on other factors. Transport cannot be reliably inferred solely using phospho-specific antibodies on whole-cell lysates.

## Introduction

Na^+^-K^+^-2Cl^−^ cotransporters are major routes for transepithelial movements of Na^+^ and Cl^−^ ions and consequently drive water flow while K^+^ is often recycled [1], [2]. There are two major isoforms, NKCC1 and NKCC2, which are products of different genes (*SLC12A2* and *SLC12A1* respectively). Both are potently and selectively inhibited by the loop-diuretic bumetanide which can be used to identify and characterise transport. NKCC1 is found mainly in the basolateral membranes of secretory epithelia where it facilitates the entry of Na^+^ and Cl^−^ into cells from interstitial fluid. NKCC1 is also widely expressed in non-epithelial cells where it helps regulate cell composition and volume. NKCC2 is found specifically in the apical membranes and subcellular vesicles of cells in the thick ascending limb of Henle's loop (TAL) in the kidney. Here, as three splice variants (NKCC2A, -B and -F), it reabsorbs about 20% of filtered NaCl from the urine, with NKCC2A distributed throughout the whole TAL. The regulation of NKCC1 has been extensively studied. Under conditions where transport is stimulated (hypertonicity, low cell [Cl^−^]) the cotransporter becomes phosphorylated on three threonine residues (equivalent to T204, T209, and T222 in ferret NKCC1 (fNKCC1)) in a key regulatory domain in the N-terminus of the transporter [3], [4]. Other studies show that phosphorylation of the cotransporter by Ste20-related proline-alanine-rich kinase (SPAK) and oxidative-stress response 1 (OSR1) kinase, is also critical in transporter activation [5]–[7], and in this case, phosphorylation of an over-lapping group of threonine residues (equivalent to T195, T199 and T204 in fNKCC1) has been demonstrated [8], [9]. Recent studies show that phosphorylation of similar well conserved residues in the N-terminus of NKCC2 (S91, T95, T100, T105 and T118 in both human and ferret NKCC2 (fNKCC2)) plays a key role in regulating activity of this transporter too [10]–[12]. Once again SPAK phosphorylates some of the residues [13]. These latter findings are an important advance in understanding the regulation of NKCC2, the study of which has been hampered by its highly restricted natural expression, and by difficulties in stably expressing the transporter in mammalian cell cultures [14]–[16].

In this paper we focus on the relationship between phosphorylation and transport rate and the effects of ouabain on transport. Given the problems of working with NKCC2, the ability to infer its activity from measures of protein phosphorylation using suitable antibodies and thus obviating the need for technically demanding transport studies, would be of immense practical benefit in studying kidney function, for instance its role in essential hypertension. However, for NKCC1 there is evidence that some factors, for instance those which alter its interactions with the cytoskeleton, may influence transport rate independently of cotransporter phosphorylation [17]–[19]. It is therefore necessary to establish whether phosphorylation of NKCC2's N-terminus is a reliable index of transport rate. In addition we explore the effects of ouabain, which is usually added during the measurement of cotransporter fluxes to reduce background fluxes through the Na^+^ pump.

Ferrets are used to model human cardiovascular physiology, for instance study of NKCC1 in ferret erythrocytes has facilitated the understanding of the transporter [18]–[20]. Previously we expressed NKCC2 cloned from ferret kidney in HEK-293 cells [16]. All three isoforms (A, B and F) were transiently expressed and carried out significant bumetanide-sensitive transport. We now report the functional expression of fNKCC1 and by comparing the properties of fNKCC1 and fNKCC2A when stably expressed in HEK-293 cells we show that, despite close similarities between these transporters, especially within the regulatory domains in their N-termini, and the common cellular environment in which they are expressed, these transporters respond differently to several stimuli. Importantly, phosphorylation does not always predict cotransport activity and ouabain strongly activates both NKCC1 and NKCC2.

## Materials and Methods

Chemicals were of analytical grade, and water was of Milli-Q quality (Millipore). Drugs were prepared as 100× stock solutions. 4-amino-5-(4-methylphenyl)-7-(t-butyl)pyrazolo[3,4-d]pyrimidine (PP1, Enzo Life Sciences, Farmingdale, NY), genistein (Sigma-Aldrich), calyculin A (Enzo Life Sciences) were dissolved in DMSO (Merck) whereas ouabain (Sigma-Aldrich) and bumetanide (Leo Laboratories, Ballerup, Denmark) were dissolved in water. Stock (200 mM) Na^+^ orthovanadate (Sigma-Aldrich) was prepared as described elsewhere [21]. ^86^RbCl was from Perkin-Elmer. Incubation media pH was adjusted to 7.4 with NaOH at the appropriate temperature.

Compositions (in mM) of media were as follows. Basic (isotonic) medium: 135 NaCl, 2.5 KCl, 1 CaCl_2_, 1 MgCl_2_, 5 glucose, 15 Hepes, pH 7.4. Hypotonic, low [Cl^−^] medium: 67.5 Na^+^ gluconate, 2.5 K^+^ gluconate, 0.5 CaCl_2_, 0.5 MgCl_2_, 5 glucose, 15 Hepes, pH 7.4. Uptake medium: 135 NaCl, 1 RbCl, 1 CaCl_2_, 1 MgCl_2_, 5 glucose, 10 sorbitol, 0.1 ouabain, 15 Hepes, pH 7.4 and ∼1 µCi/ml ^86^Rb^+^. Lysis buffer: 50 NaF, 5 Na_4_P_2_O_7_, 5 EDTA, 1 Na^+^ orthovanadate, 1% Triton X-100, 1% protease inhibitor cocktail (Merck), and 20 Hepes, pH 7.4. Na^+^-free medium was similar to basic medium but with all Na^+^ replaced by N-methyl-d-glucamine (NMDG), and Hepes neutralised with Tris-base.

### RNA isolation and Cloning of ferret NKCC1

Total RNA preparation from ferret kidneys and subsequent cloning of the 5′- and 3′- regions of the fNKCC1 gene were performed as described earlier [16] using fNKCC1 gene-specific primers based on conserved regions of known vertebrate NKCC1 gene sequences. Using the sequence data obtained, the complete open reading frame was amplified from random hexamer and oligo-dT transcribed ferret kidney cDNA pools as two fragments coding for either the N-terminus or C-terminus of fNKCC1, each encompassing a unique *Bam*HI restriction site in the second half of the fNKCC1 gene (N-terminus: 5′-CTG CCG GGG GTA CCG CAG CGA TGG AAC C-3′, 5′-ATC GAA TGT TGC AGT GCA TTT AGG-3′; C-terminus: 5′-GGT AAT GAG CAT GGT GTC AGG A-3′, 5′-CAT GAG GGC TGT CCA GAA TTC AGG GCC TTT AAG-3′), and cloned into the pGEM-T-Easy vector (Promega) to give pGEM-5′-f1 and pGEM-3′-f1, respectively. The 5′- and 3′-NKCC1 fragments were subcloned into pcDNA3.1 using a three way ligation to obtain the complete open reading frame: pGEM-5′-f1 was digested with *Kpn*I/*Bam*HI and pGEM-3′-f1 with *Bam*HI/*Eco*RI and the resulting fragments were ligated into the *Kpn*I/*Eco*RI sites of pcDNA3.1. From that, the complete fNKCC1 coding region was amplified using 5′-GCT GGC TAG CGT TTA GAA TTC AGC TTG GTA CCG CAG CG-3′ and 5′-TAG AAG GCA CAG TCG AGG-3′, inserted into the *Eco*RI site of pCI-neo (Promega) and the obtained pCI-fNKCC1 construct sequenced (CoGenics, Essex, UK).

Sequence data for fNKCC1 was deposited at GenBank under accession number GQ338078.

### Cell culture and stable cell lines

HEK-293 cells were maintained in Dulbecco's Modified Eagle Medium (DMEM) supplemented with 10% heat-inactivated fetal bovine serum, 100 U/ml penicillin, 100 µg/ml streptomycin, 4 mM L-glutamine and, in case of stably transfected cells, 0.15 mg/ml Geneticin (Invitrogen). All cells were maintained at 37°C in a water-saturated atmosphere containing 95% air and 5% CO_2_. To create a cell line stably expressing fNKCC1, HEK-293 cells were transfected with pCI-fNKCC1 using ExGen 500 (Fermentas, Ontario, Canada) according to the manufacturer's instructions and stable transfectants were selected by growth in the presence of 0.3 mg/ml Geneticin. HEK-293 cells stably expressing fNKCC2A have been described before [16]. Cells were plated onto poly-d-lysine coated 96-well plates and grown to confluence for 2–3 days before experimentation.

### ^86^Rb^+^ uptake studies

NKCC activity was measured as bumetanide-sensitive ^86^Rb^+^ influx in wild-type or stably transfected HEK-293 cells grown to confluence in 96-well plates as described previously [16]. Fluxes were measured at room temperature (RT; 20–25°C) or at 37°C. Cells were washed and briefly incubated in basic medium, followed by a three-step protocol. In step 1, the cells were incubated for a total of 60 min in a pre-incubation medium. This was either basic medium or a hypotonic, low [Cl^−^] medium. Drugs were added to basic medium during this period. Where used, orthovanadate (1 mM) was present for the full 60 min whereas DMSO (1%), calyculin A (0.25 µM), PP1 (50 µM), and genistein (120 µM) were present for the final 30 min. In experiments on the time-dependence of the effects of ouabain or Na^+^-removal, ouabain (0.1 mM) or the Na^+^-free medium was present for the last 5–30 min. In step 2 cells were incubated for 5 min in the final pre-incubation medium (containing any drugs) or the Na^+^-free medium with the addition of 0.1 mM ouabain. In step 3 ^86^Rb^+^ uptake was measured in uptake medium containing any drugs where indicated. Uptake was measured for 2–3 min at RT, for 1 min at 37°C, and for 7 min when bumetanide was present. In all cases the bumetanide-resistant uptake was measured using an identical protocol but with 10 µM bumetanide present during steps 2 and 3. ^86^Rb^+^ uptake was terminated by removing the uptake medium and washing cells three times in cold 110 mM MgCl_2_ using an automatic plate-washer (Multiwash III; TriContinent, Grass Valley, CA). Cell ^86^Rb^+^ was determined with a Typhoon phosphor imager (GE Healthcare). At least 3 wells were used for each experimental point. Cell protein content for each construct was determined from wells which had not received ^86^Rb^+^. Cells were lysed in lysis buffer and protein concentration was determined using a BCA protein assay (Pierce, Rockford, IL).

### Phosphorylation of NKCC, SDS-PAGE and Antibodies

Wild-type and stably transfected HEK-293 cells were incubated at 37°C as for Rb^+^ uptake assays described above, but without addition of ^86^Rb^+^. After removal of uptake medium, the plates were immediately placed on ice and cells were lysed in ice-cold lysis buffer. Lysates from four wells were combined and their protein content determined. SDS-PAGE and Western blotting were performed as described previously [16]. Primary antibodies were: N1 (0.5 µg/ml [16]), T4 (1∶10000; Developmental Studies Hybridoma Bank, Iowa City, IA), R5 (1∶10,000; gift of B Forbush, Yale), NKCC-P and anti-NKCC2 (1 µg/ml and 0.25 µg/ml; gifts of DR Alessi, Dundee, UK); HRP-conjugated secondary antibodies were: rabbit anti-sheep (1∶60,000; Pierce) and goat anti-rabbit or anti-mouse (1∶20,000; Jackson ImmunoResearch). N1 is a specific anti-NKCC1 rabbit polyclonal antibody raised to two peptides in the N-terminus of human NKCC1 [16], T4 is a mouse monoclonal antibody that binds to the C-terminus of both NKCC1 and NKCC2 [22], and anti-NKCC2 is a sheep polyclonal antibody raised to residues 1–174 in the N-terminus of human NKCC2 [13]. R5 was raised to a di-phosphopeptide (containing the equivalents of T204 and T209 in fNKCC1, or T100 and T105 in fNKCC2A) and reacts with threonine residues in NKCC1 and NKCC2 that are phosphorylated when the transporter is activated by exposure to low cell [Cl^−^] [11], [23], whereas NKCC-P reacts with the three threonine residues phosphorylated by SPAK/OSR1 [8], which are equivalent to T195, T199, T204 in fNKCC1 or S91, T95, T100 in fNKCC2A. Equal loading of samples was confirmed by staining gels for protein (GelCode Blue, Pierce) and comparing the optical densities of bands using TotalLab (Non Linear Dynamics, Newcastle, UK). A biotinylated protein ladder (Cell Signaling Technology, Danvers, MA) was used for estimation of molecular mass.

### Statistical analysis

Values are given as means ± s.e.m. with n the number of independent experiments. Occasionally, it was not possible to repeat every component of an experiment because of cell instability. Data analysis under these circumstances is described in figure legends. Differences between normalised means were assessed using two-tailed t-tests, with the level for significance set at P<0.05. Non-linear regression analysis (one-phase association, no weighting) was performed using Graph Pad Prism (version 5.0, Graph Pad Software, San Diego, CA).

## Results

### Stable expression of fNKCC1

**Figure 1:**
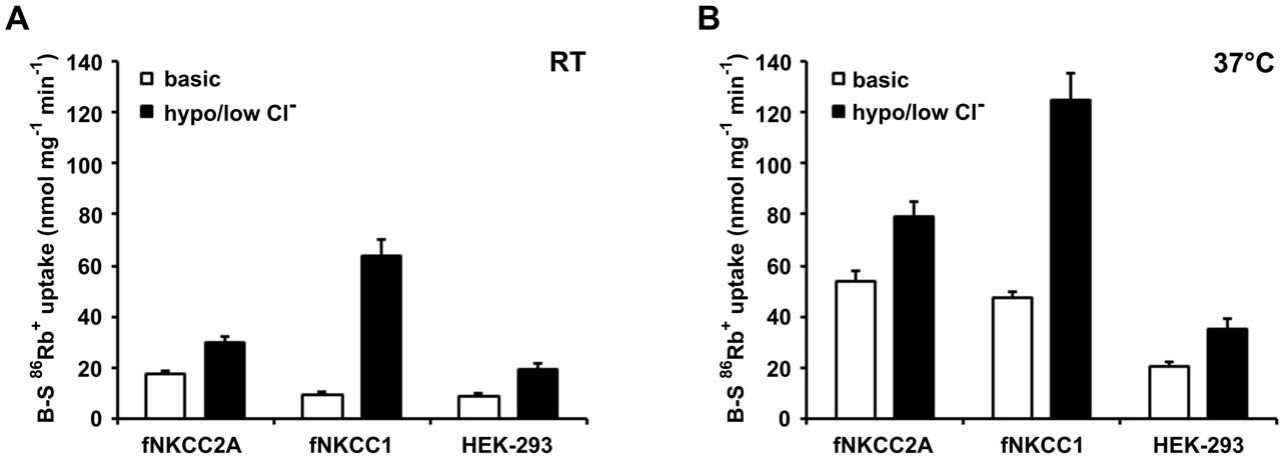
Temperature-dependence of ^86^Rb^+^ uptake. ^86^Rb^+^ uptake in the absence or presence of 10 µM bumetanide was assessed following a 60 min pre-incubation in isotonic basic (*open bars*) or hypotonic, low [Cl^−^] (*filled bars*) medium at either RT (A) or 37°C (B). Bumetanide-sensitive (B-S) ^86^Rb^+^ uptakes are shown as mean ± s.e.m. (n = 9–14). In experiments designed specifically to assess the effects of increasing the temperature from RT to 37°C, ^86^Rb^+^ uptake increased by: fNKCC2A, 343±13% (n = 4); fNKCC1, 509±22% (n = 3); and HEK-293, 150±49% (n = 3) under basal conditions; and by: 236±36% (n = 4), 106±17% (n = 5), and 145±31% (n = 3) following incubation in a hypotonic, low [Cl^−^] medium. Bumetanide-resistant ^86^Rb^+^ uptakes at 37°C in basic medium were (nmol mg protein^−1^ min^−1^): fNKCC2A, 1.86±0.77 (n = 10); fNKCC1, 1.95±0.37 (n = 10); and HEK-293, 1.04±0.39 (n = 8) and did not change substantially under any condition tested.

**Figure 2:**
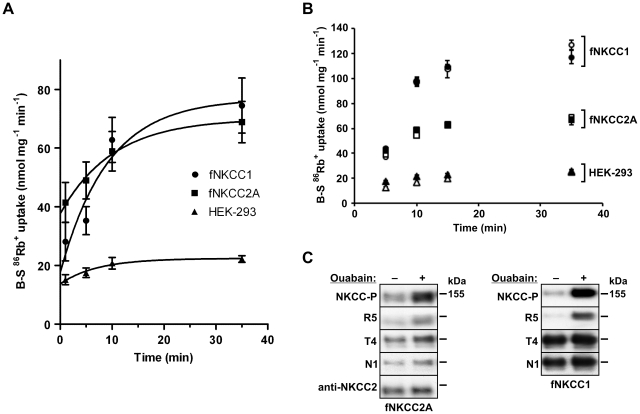
Effect of ouabain on ^86^Rb^+^ uptake and phosphorylation of fNKCC2A and fNKCC1. (A) time-dependent activation of ^86^Rb^+^ uptake by ouabain. Bumetanide-sensitive (B-S) ^86^Rb^+^ uptakes were measured at 37°C following pre-incubation of cells in an isotonic medium containing 0.1 mM ouabain for the times indicated. Values are means ± s.e.m. (n = 4–7). *Circles* represent fNKCC1; *squares*, fNKCC2A; and *triangles*, control HEK-293 cells. Lines, fitted to the data by non-linear regression analysis, are of the form: y = R+S(1−exp(−kt)), where R is the initial, and R+S the maximum uptake, k is the rate constant and t, the time in min. (B) Effect of omitting Ca^2+^ from the medium during exposure to ouabain. Cells were exposed to 0.1 mM ouabain and ^86^Rb^+^ uptake was measured in the presence (*open symbols*) or absence (*filled symbols*) of 1 mM Ca^2+^ in the medium. Ca^2+^-chelators were not used. (C) Lysates from cells exposed to ouabain (in the presence of Ca^2+^) for 30 min were immunoblotted and probed with antibodies against phospho-NKCC1 and 2 (NKCC-P, R5), NKCC2 (anti-NKCC2), NKCC1 and 2 (T4) and NKCC1 (N1). An indication of molecular mass is shown to the right of each panel. Similar results were obtained when the experiment was repeated.

Ferret NKCC1 was cloned from adult kidney. The expressed protein comprises 1204 amino acids with a calculated molecular mass of 131 kDa (unglycosylated) and a pI of 6.03. The protein has 95% and 94% similarity with human and mouse NKCC1 respectively. The threonine residues in the N-terminus regulatory domain are conserved together with the surrounding sequence. Also in the N-terminus are two consensus sites (^80^RFQV and ^130^RFRV) for binding SPAK and OSR1 [5] and one site (^132^RVNF) for binding protein phosphatase 1 (PrP1) [24] which partially overlaps with the second SPAK/OSR1 binding domain. In SDS-PAGE, the full-length glycosylated protein migrates as bands at approximately 155 kDa (Figure 2C) and also at 316 kDa. When stably expressed in HEK-293 cells (fNKCC1 cells) cotransport activity under isotonic conditions at RT was not significantly different from that in untransfected cells (Figure 1A). Incubation of fNKCC1 cells for 60 min in a hypotonic, low [Cl^−^] medium prior to flux measurement (in an isotonic medium) caused a large activation of transport (Figure 1A), similar to that seen with cells transfected with shark or human NKCC1 [25]. Activation results from the large fall in cell [Cl^−^] and the subsequent cell shrinkage on return to an isotonic medium. This procedure is frequently used to maximally activate NKCC1 [3], [25] or NKCC2 [12]. It also increased phosphorylation of the N-terminus regulatory domain of fNKCC1 as detected by two different phospho-specific antibodies, R5 and NKCC-P (Table 1). Insertion of fNKCC1 into the surface membrane was confirmed by its detection amongst those proteins pulled down by streptavidin beads following exposure of cells to a membrane-impermeant biotinylation reagent (not shown; for methods see [16]).

**Table 1:** Effects of inhibitors and changes to the ionic environment on cotransporter phosphorylation.

	Hypo/low	Ouabain^a^	Na^+^-free^b^	Calyculin	PP1	Genistein
	[Cl^−^]	0.1 mM		0.25 µM	50 µM	120 µM
fNKCC2A cells^c^						
−134 kDa	2.3±0.3	1.9±0.3	1.3±0.1	1.6±0.1	0.4±0.1	0.5±0.1
−274 kDa	2.8±0.7	3.0±1.0	1.6±0.2	4.3±1.2	0.5±0.1	0.2±0.1
−155 kDa	2.4±0.5	2.2±0.4	1.3±0.1	5.3±2.0	0.3±0.1	0.5±0.1
fNKCC1 cells						
−155 kDa	2.5±0.9	3.9±1.6	2.1±0.6	5.6±2.1	0.5±0.1	0.7±0.2
HEK-293 cells						
−155 kDa	1.4	1.5	1.4±0.4	2.7±1.0	0.6±0.1	0.6±0.1

Phosphorylation levels detected by antibodies R5 and NKCC-P expressed as factors of appropriate controls. Values are given as mean ± s.e.m. (n≥4) or as averages if n = 2.

a Cells were exposed to ouabain for 35 min.

b Cells were exposed to Na^+^-free medium for 5 min.

c 134 kDa and 274 kDa bands represent monomeric and dimeric fNKCC2A; 155 kDa bands, endogenous NKCC and fNKCC1. As antibodies R5 and NKCC-P detect similar changes in cotransporter phosphorylation data have been pooled.

### Effect of temperature

Na^+^-K^+^-2Cl^−^ cotransport is often measured in transfected cells by growing them at 37°C in culture medium, then washing them in a simplified buffer solution containing 0.1 mM ouabain, before measuring K^+^ (^86^Rb^+^) uptake in the presence of ouabain. The latter steps are usually carried out at RT [16], [25] which slows uptake to allow more precise measurements. At RT (Figure 1A, open bars), after isotonic pre-incubation bumetanide-sensitive ^86^Rb^+^ uptake by fNKCC1 cells was indistinguishable from that by control HEK-293 cells, whereas it was twice as high in HEK-293 cells expressing fNKCC2A (fNKCC2A cells). Exposure to a hypotonic, low [Cl^−^] medium was needed to distinguish fNKCC1 from HEK-293 NKCC (Figure 1A, filled bars). On raising the temperature to 37°C cotransporter fluxes under isotonic conditions were far larger in fNKCC1 and fNKCC2A cells than in HEK-293 cells (Figure 1B, open bars). The rise in temperature clearly reveals the presence of the transfected cotransporters, particularly fNKCC1. Pre-incubation in a hypotonic, low-[Cl^−^] medium increased uptake in all three cell types at 37°C but the effects were smaller than at RT (Figure 1B, filled bars). Although we can calculate an approximate value for the temperature-dependence from these data, more precise estimates require correction for the effects of ouabain as described in the next section.

### Effect of ouabain

In the previous experiments cells were incubated with ouabain for 5 min before flux measurement. The time was chosen to facilitate addition of medium to the 96-well plates and to ensure that all samples were treated similarly. However, we were concerned that cell ion contents could change significantly when cells were incubated with ouabain at 37°C and thereby influence fluxes. Figure 2A shows the effects of changing the duration of exposure to ouabain on the cotransport fluxes at 37°C. Bumetanide-sensitive ^86^Rb^+^ uptake increases substantially with exposure time in fNKCC1 cells, but increases far less in HEK-293 cells and fNKCC2A cells. In order to assess whether Ca^2+^ entry via an active Na^+^/Ca^2+^ antiporter was responsible for the stimulation of cotransport the experiments were repeated in the absence of added external Ca^2+^. Figure 2B shows that cotransport stimulation was identical in the absence of external Ca^2+^ ruling out a role for Ca^2+^ entry in this particular response.

Incubation of cells for 30 min with ouabain caused substantial increases in the phosphorylation of fNKCC1 (4-fold) and fNKCC2A (2–3 fold), and a smaller increase in that of endogenous HEK-293 NKCC as detected by phospho-specific antibodies R5 and NKCC-P (Figure 2C, Table 1). These data suggest that at least part of the responses to ouabain is due to increased transporter phosphorylation, and not simply due to changes in the concentrations of the cotransporter's substrate ions.

Extrapolation of data in Figure 2A suggest that if the addition of ouabain could have been made instantaneously the fNKCC1 and fNKCC2A fluxes would have been 50 and 81% of the fluxes measured at 5 min respectively. Correcting the data obtained at 37°C in Figure 1B for the effects of ouabain (it does not affect cotransporter fluxes at RT, data not shown), the temperature dependencies, expressed as Q_10_, of fNKCC1 and fNKCC2A fluxes are 2.18 and 2.15 respectively. Data in Figure 2A also indicate that the maximal rates seen after prolonged incubation in ouabain are about 63% (HEK-293 and fNKCC1) or 90% (fNKCC2A) of the values seen following pre-incubation in a hypotonic, low [Cl^−^] medium (Figure 1B, filled bars).

### Incubation in a Na^+^-free medium

**Figure 3:**
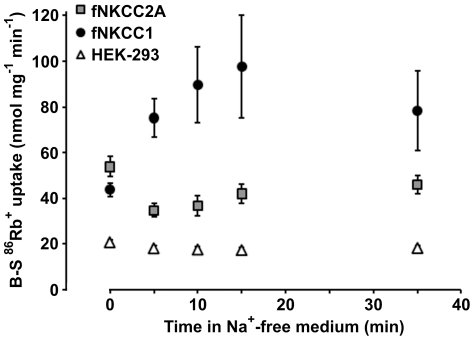
^86^Rb^+^ uptake after pre-incubation in Na^+^-free medium. Bumetanide-sensitive (B-S) ^86^Rb^+^ uptake was measured at 37°C in confluent HEK-293 cells (*triangles*), fNKCC2A cells (*squares*) or fNKCC1 cells (*circles*). Cells were pre-incubated in a Na^+^-free (NMDG) medium for the times indicated with 0.1 mM ouabain present for the last 5 min. ^86^Rb^+^ uptake was then measured in the presence of Na^+^. Points represent means ± s.e.m. (n = 5–11).

Given that prolonged ouabain exposure, a manoeuvre that raises cell [Na^+^], activated both NKCC1 and NKCC2A we next examined the consequences of pre-incubating cells in a nominally Na^+^-free (NMDG) medium (Figure 3). This caused a slight fall in the activity of endogenous HEK-293 NKCC whereas the activity of fNKCC1 more than doubled in 15 min, reaching values close to those seen following incubation in a hypotonic, low [Cl^−^] medium. fNKCC2A activity on the other hand fell sharply by 34% within 5 min and then slowly increased over the following 30 min but did not fully return to its initial value. These changes in transport were accompanied by changes in cotransporter phosphorylation measured 5 min after exposure to the Na^+^-free medium (Table 1). Phosphorylation of fNKCC1 approximately doubled whereas that of the endogenous NKCC in HEK-293 and fNKCC2A cells (155 kDa band), and of fNKCC2A increased by 30–60%.

### Effects of kinase and phosphatase inhibitors on ^86^Rb^+^ uptake

**Figure 4:**
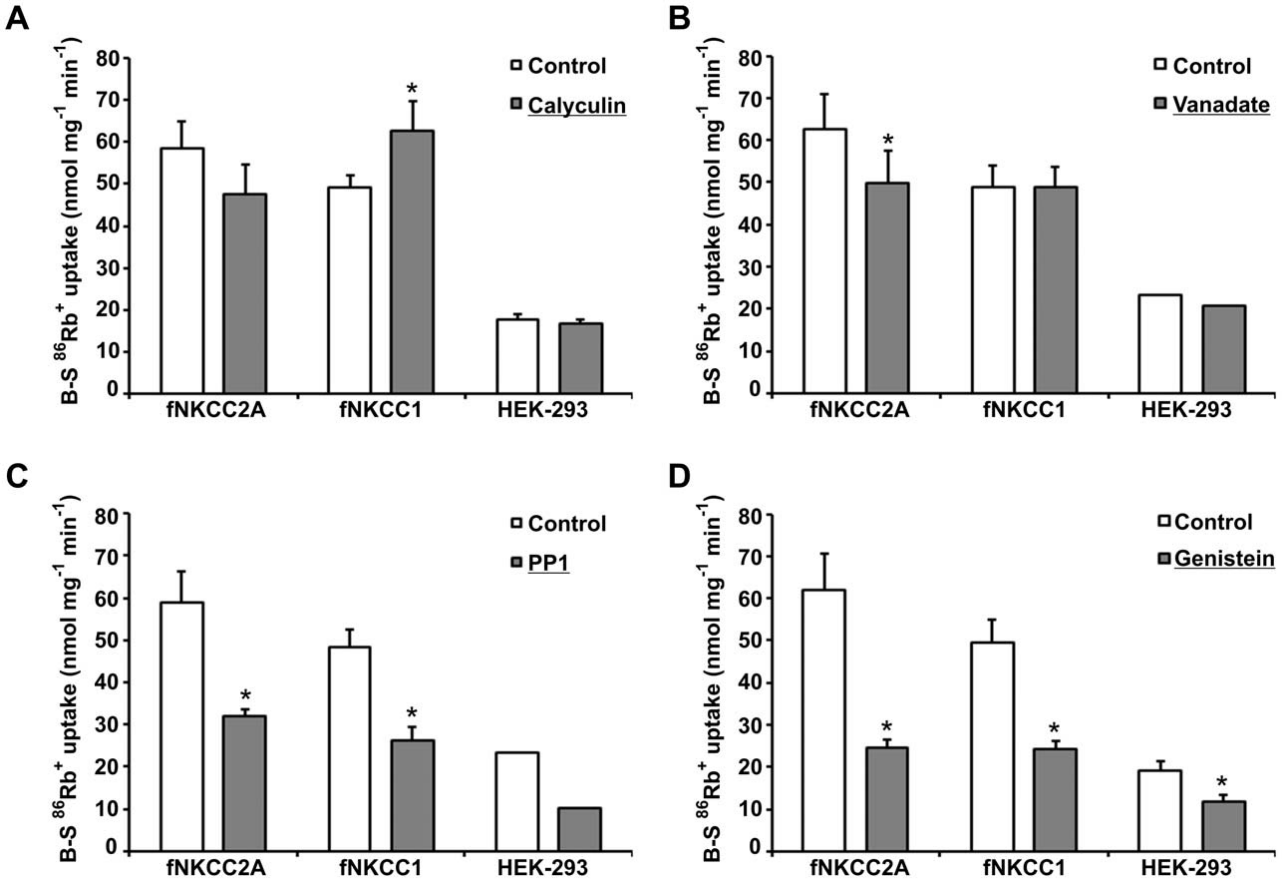
Effects of phosphatase and kinase inhibitors on ^86^Rb^+^ uptake. Cells were pre-incubated at 37°C in basic medium for 30 min followed by 30 min in basic medium in the absence (control) or presence of: 1% DMSO (vehicle), 0.25 µM calyculin (A), 1 mM orthovanadate for the entire 60 min (B), 50 µM PP1 (C) or 120 µM genistein (D) before measuring ^86^Rb^+^ uptake in the presence of the drugs. Bumetanide-sensitive (B-S) ^86^Rb^+^ uptakes are shown as mean ± s.e.m. (n = 3–7, values for HEK-293 cells in *B* and *C* are means of triplicates in a single experiment). Using paired, normalised comparisons * signifies P<0.05. ^86^Rb^+^ uptakes in the presence of DMSO were (% control): fNKCC2A, 91.7±2.4 (n = 6); fNKCC1, 93.0±5.2 (n = 6); and HEK-293, 101±5.7 (n = 3), none significantly different from controls.

As regulation of NKCC activity is linked to phosphorylation of key threonine residues in the N-terminus of the cotransporter we tested the effect of different phosphatase and kinase inhibitors. Concentrations and exposure times were chosen on the basis of previous experience of these reagents on transport in other cells [20], [25], and to minimize deleterious effects on HEK-293 cell viability. 0.25 µM Calyculin A (Figure 4A), a potent inhibitor of protein phosphatases 1, 2A, 4, 5 and 6, which stimulates ^86^Rb^+^ uptake in cells transfected with human NKCC1 [6], and in erythrocytes [20], [21] and shark rectal glands [3] had no effect on bumetanide-sensitive ^86^Rb^+^ uptake in untransfected HEK-293 cells (95.7±3.9% control, n = 4), whereas it increased fluxes by 33.4±4.8% (n = 6, P = 0.001) in fNKCC1 cells, but slightly reduced fluxes by 18.9±10.3% (n = 7, P = 0.116) in fNKCC2A cells. Although calyculin A increased fNKCC1 activity this represents only about 15% of the stimulation seen when cells are pre-incubated in hypotonic low [Cl^−^], or low [Na^+^] media.

Experiments on rat parotid acinar cells showed that Na^+^ orthovanadate, an inhibitor of tyrosine phosphatases (as well as a potent inhibitor of P-Class ATPases such as the Na^+^ and Ca^2+^ pumps), markedly inhibits the dephosphorylation of NKCC1 [26]. We therefore tested the effect of 1 mM orthovanadate on bumetanide-sensitive ^86^Rb^+^ uptake (Figure 4B). It had no effect on uptake in HEK-293 (89.3±6.4% control, n = 4) and fNKCC1 (100.2±3.6%, n = 4) cells, but significantly inhibited ^86^Rb^+^ uptake in fNKCC2A cells by 20.6±2.1% (n = 4; P = 0.0023). In contrast to these differential effects of phosphatase inhibitors, treatment of cells with kinase inhibitors, 50 µM PP1 or 120 µM genistein, reduced ^86^Rb^+^ uptake in all three cell types by approximately 50% (Figure 4C and D).

### Effects of kinase and phosphatase inhibitors on NKCC phosphorylation

**Figure 5:**
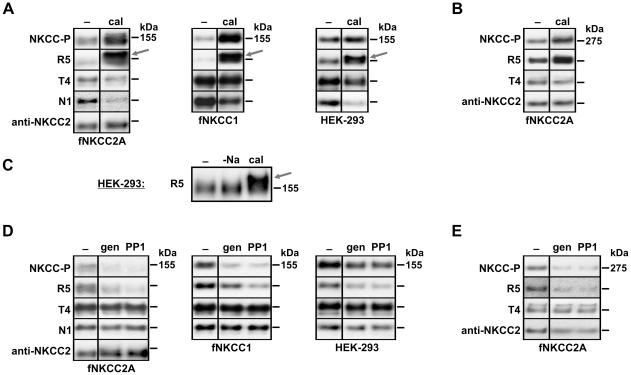
Effects of phosphatase and kinase inhibitors on phosphorylation of fNKCC2A and fNKCC1. Experiments were carried out as described in Figure 4, but without the addition of ^86^Rb^+^. Cell lysates were subjected to SDS-PAGE, immunoblotted, and probed with antibodies against phospho-NKCC1 and 2 (NKCC-P, R5), NKCC2 (anti-NKCC2), NKCC1 and 2 (T4) and NKCC1 (N1). Representative blots are shown with control and drug-treated samples from the same blot. Estimated molecular mass is indicated to the right of each panel. (A) Effect of 0.25 µM calyculin A on monomeric NKCC. An approximate 20 kDa band-shift in calyculin-treated cells is only observed with the phospho-specific antibody R5 (*arrow*) but not with NKCC-P. (B) High molecular mass bands in calyculin A treated fNKCC2A cells. (C) Example of 20 kDa band-shift (*arrow*) in HEK-293 cells. Cells had been pre-incubated in basic medium, a Na^+^-free medium or with calyculin. (D) Effect of 50 µM PP1 and 120 µM genistein on monomeric NKCC. (E) High molecular mass bands in PP1 or genistein treated fNKCC2A cells.

Phosphorylation was assessed using R5 and NKCC-P after cells had been treated with 0.25 µM calyculin A, 50 µM PP1 or 120 µM genistein at 37°C (Figure 5). In fNKCC2A cells, NKCC2-specific bands were detected at ∼134 kDa and at ∼274 kDa (possible dimer) in addition to endogenous NKCC at 155 kDa. In HEK-293 and fNKCC1 cells there was one major band at ∼155 kDa, comprising endogenous NKCC alone or with fNKCC1. A minor band at ∼316 kDa was occasionally observed with T4 (NKCC1 dimer). As R5 and NKCC-P reported similar changes in the phosphorylation of either cotransporter under all conditions, phosphorylation data have been pooled in Table 1.

Treatment with calyculin A led to a 3–5-fold increase in phosphorylation of endogenous NKCC (in HEK-293 and fNKCC2A cells) and a 5-fold increase in fNKCC1 phosphorylation observed at 155–175 kDa (Figure 5A, Table 1). In almost all experiments, and with all three cell types, the majority of the R5 band at 155 kDa was replaced by a more slowly migrating band at ∼175 kDa (Figure 5A and C, arrows). This ∼20 kDa band-shift was accompanied by a reduction in the intensity of the 155 kDa band detected with NKCC1-specific antibody N1 (fell to 28±5.9% control, n = 5) and, to a smaller extent with T4 (fell to 62±9.5% control, n = 5). In the few cases where the band-shift was not seen, there was no change in NKCC1-detection with N1 or T4. fNKCC2 bands at both 274 and 134 kDa also showed increased phosphorylation (4-fold and 60% respectively) after calyculin A treatment (Figures 5A and B), but in this case there were no band-shifts and nor were there changes in the intensities of bands detected with anti-NKCC2 or T4 (Figure 5A and B).

Overall there was a poor correlation between changes in phosphorylation and transport in the presence of calyculin A. In HEK-293 cells increased phosphorylation of endogenous NKCC was not accompanied by any significant change in cotransporter fluxes, and in fNKCC1 cells, a five-fold increase in phosphorylation was accompanied by only a 30% increase in transport (Figure 4A). For fNKCC2A a significant rise in phosphorylation was accompanied by a small drop in transport (Figure 4A). However, the kinase inhibitors PP1 (50 µM) and genistein (120 µM) reduced phosphorylation of fNKCC1 and fNKCC2A by about 50% (Figure 5D and E, Table 1) similar to the reduction they caused in activity (Figures 4C and D). Total NKCC1 and NKCC2 protein levels remained constant throughout these experiments (Figure 5D and E).

## Discussion

We describe the properties of fNKCC1 stably expressed in HEK-293 cells and compare them with those of stably-expressed fNKCC2A (62% sequence similarity), the NKCC2 isoform which is present throughout the TAL and has similar ion affinities to NKCC1 [27]. Fluxes through fNKCC1 and fNKCC2A could be clearly distinguished from those through the endogenous HEK-293 NKCC by making the measurements at 37°C. Similar high rates of transport (100–130 nmol mg^−1^ min^−1^) were observed in fNKCC1 cells following their pre-incubation in a hypotonic, low [Cl^−^] medium (Figure 1B), and surprisingly after preincubation in a Na^+^-free medium (Figure 3), and may represent the maximum achievable. fNKCC1 thus runs at about 20% maximum at 37°C, similar to the level seen with the endogenous transporter in erythrocytes [20]. The very low activity of fNKCC1 at RT (Figure 1A) may result from the slight cell swelling and take up of Cl^−^ and Na^+^ that occurs when cells are cooled. Even though the largest fluxes through fNKCC2A were observed following pre-treatment of cells in a hypotonic, low [Cl^−^] medium, fNKCC2A appears less sensitive to changes in cell [Cl^−^] than fNKCC1 (Figure 1B). Indeed we generally observed that, with fixed external substrate concentrations, many perturbations affected the activity of fNKCC2A far less than that of fNKCC1. However, treatment of cells with ouabain stimulated fNKCC2A (Figure 2) to about 90% of its highest level, whilst it stimulated fNKCC1 to about 63% of its maximal rate.

The links between phosphorylation of two neighbouring domains in the N-termini of fNKCC1 and fNKCC2A and transport rate (activity) were examined. Treatment of cells in a variety of ways can produce similar patterns of phosphorylation, but it is often difficult to assign these effects appropriately to those kinases or phosphatases that phosphorylate or dephosphorylate the cotransporter itself as kinase activation and phosphatase inhibition have similar effects. What clearly emerges from our studies is that inhibition of kinases reduces phosphorylation with proportional changes in transport, and that transport stimulation is only seen when phosphorylation increases from its basal level. However, it does not always follow that an increase in phosphorylation results in transport stimulation. Phosphorylation is necessary, but not sufficient. Thus phospho-specific antibodies may provide important information about cotransporter phosphorylation and the integrity of cell signalling pathways but they may not be as reliable for reporting cotransporter activity.

Both PP1, a kinase inhibitor selectively affecting Src, Lck, RIP2, GAK, and CK1δ [28], and genistein, a less selective inhibitor, reduce the resting activities of fNKCC1, fNKCC2A and the endogenous HEK-293 NKCC by about 50% (Figures 4C and D), very similar to the effects seen in erythrocytes [19]–[21]. These reductions in transport were accompanied by similar reductions in cotransporter phosphorylation (Figures 5D and E, Table 1). Just as with erythrocytes it was not possible to inhibit all ^86^Rb^+^ uptake or cotransporter phosphorylation using kinase inhibitors [18], perhaps suggesting that persistent basal phosphorylation and activity is a feature of these cotransporters. Although both genistein and PP1 are often considered to be tyrosine kinase inhibitors, changes in the tyrosine phosphorylation of NKCC1 which relate to changes in transport rate have not been observed using either phospho-peptide mapping [3] or phospho-specific antibodies [29]. Both inhibitors have broader specificity [28] so whether genistein or PP1 inhibit the cotransporter kinases directly or via up-stream kinases remains uncertain.

Incubation of cells in a hypotonic, low-[Cl^−^] medium (Figure 1) or with ouabain (Figure 2) activates both fNKCC1 and fNKCC2A which is accompanied by 2–4 fold increases in their phosphorylation (Table 1). The effects of hypotonic, low-[Cl^−^] pre-incubation are caused, at least in part, by the activation of SPAK/OSR1 [6], [9], [30], kinases that phosphorylate and activate the transporters. However, the strong stimulation of fNKCC1, fNKCC2A and endogenous NKCC by ouabain was not anticipated, though it transpires that stimulation of loop-diuretic-sensitive K^+^ uptake (presumably via NKCC1) by ouabain had been reported in cultured cells [31], [32]. We ruled out that it was secondary to a rise in cell [Ca^2+^] as the same responses were seen in cells incubated in Ca^2+^-free media (Figure 2B), and nor did it stem from the rise in cell [Na^+^], or fall of cell [K^+^], as vanadate, another potent Na^+^-pump inhibitor which should have similar effects on cell ion contents, had no effect on fNKCC1 whilst it slighted inhibited fNKCC2A (Figure 4B). An alternative possibility is that ouabain itself, by binding to the pump, disturbs its interaction with Src kinase, and thus activates signalling pathways that affect many cell processes [33], including cotransporter activity [18], [20]. These findings may be physiologically significant as the levels of endogenous ouabain-like compounds fluctuate in the circulation [34] and could thus affect Na^+^ reabsorption in the TAL. Our findings also suggest that care must be taken when using ouabain during measurement of cotransporter fluxes.

Several treatments had opposite effects on transport mediated by either fNKCC1 or fNKCC2A. Initially we hypothesised that these differences might result from the ability of NKCC1, but not NKCC2, to bind the catalytic subunit of PrP1 [11], [24]. This bound PrP1 could then dephosphorylate both NKCC1 itself, and SPAK/OSR1 bound to NKCC1 at an adjacent site [35]. Thus manoeuvres that affect PrP1 might change the phosphorylation and activity of fNKCC1 directly as well as indirectly by affecting the activity of SPAK/OSR1, whilst they would not be expected to affect fNKCC2A. However, measurements of phosphorylation did not support this hypothesis.

Calyculin A activated fNKCC1 and caused a slight inhibition of fNKCC2A (Figure 4A). This is consistent with the model above and with the previous observations that calyculin does not activate transport by NKCC2 expressed in *Xenopus* oocytes [12], and that it only stimulates transport in cultured TAL cells by 20% [36]. In contrast, phosphorylation of both fNKCC1 and fNKCC2A increased substantially (Figure 5, Table 1). For fNKCC2A this increase was especially unexpected given the effect on transport, but supports the idea that PrP1 bound to other chaperones, for instance apoptosis-associated tyrosine kinase, might also reduce cotransporter phosphorylation [37]. However, even for fNKCC1 a 5-fold increase in phosphorylation was associated with only a 30% increase in transport, far below the transporter's capacity to respond under other conditions (Figures 1B, 2A, 3 and Table 1). This suggests that while phosphorylation of the regulatory domains in the N-terminus determines the transporter's potential capacity to move ions, the actual activity also depends on a variety of other factors. For calyculin, which inhibits many phosphatases in addition to PrP1 (with consequent activation of kinases), we hypothesise that cotransporter hyperphosphorylation may inhibit transport. One possibility is that cotransporters have sites whose phosphorylation inhibits transport. Alternatively the effect may be indirect, for example phosphorylation of a particular site may reduce the transporters' cell surface expression, either by reducing trafficking to the plasma membrane, or by stimulating removal from the surface and possibly destruction in the proteasome. Calyculin causes large proportionate increases in phosphorylation and transport (NKCC1) in erythrocytes [21]. In these cells trafficking does not affect surface expression of transporters favouring the view that calyculin's inhibitory effects stem from it interfering with trafficking.

fNKCC1 and fNKCC2A also responded differently when cells had been pre-incubated in a [Na^+^]-free medium (Figure 3 and Table 1). fNKCC1 was almost maximally activated and its phosphorylation doubled, whereas fNKCC2A was inhibited, with perhaps a small rise in its phosphorylation. Incubation of cells in a Na^+^-free medium would be expected to deplete their intracellular [Na^+^] at a rate that depends on the Na^+^ pump and the availability of additional Na^+^ efflux routes. As transfected fNKCC1 and fNKCC2A provide such routes, intracellular [Na^+^] could fall to a low level at different rates. However, this is unlikely to cause substantial differences in cell [Na^+^] over the time course of our experiments given the contribution of the Na^+^ pump, and the similar maximal transport rates and ion affinities of the isoforms involved. Changes in cell [Na^+^] could then cause secondary changes in cell [Ca^2+^] and pH. The effects external Na^+^-removal on these variables have been documented for a few cell types, for instance cardiac myocytes [38], but is beyond the scope of the current study. Thus it is not possible to identify the precise change that causes the different responses of fNKCC1 and fNKCC2A, but it is clear that striking differences are apparent after 5 min exposure to a Na^+^-free medium and are maintained for at least the next 30 min. The simplest explanation is that reductions in cell [Na^+^] affect phosphorylation and/or trafficking of fNKCC1 and fNKCC2A differently and suggest that cell [Na^+^], in addition to being a substrate, may regulate cotransport.

Recently, NKCC1 and NKCC2F, expressed in *Xenopus* oocytes, were shown to behave differently when the medium was made hypertonic by addition of sucrose [39]. Whereas ^86^Rb^+^ uptake by NKCC1 was strongly stimulated, uptake by NKCC2F only rose slightly. In addition, the stimulated uptake through NKCC1 represented K^+^-K^+^ exchange [39], [40], perhaps facilitated by the rise in intracellular ion concentrations (particularly Cl^−^) under these conditions, and perhaps suggesting that NKCC1 is more prone to this mode of transport than NKCC2F. It is likely that some K^+^-K^+^ exchange contributes to ^86^Rb^+^ uptake measured here. However, the ion gradients favoured net influx (rather than K^+^-K^+^ exchange) under most conditions (and cell [Cl^−^] was often reduced), except perhaps after long exposure to ouabain, where a rise in intracellular [Na^+^] might favour self-exchange. Conversely, a fall in K^+^-K^+^ exchange might explain the slight drop in ^86^Rb^+^ uptake seen in HEK-293 cells incubated in a Na^+^-free medium.

The differences between fNKCC2A and fNKCC1 shown in this study - ranging from fNKCC2A's higher resting activity to its reduced responsiveness to a variety of stimuli - are in line with the different locations and physiological roles of the transporters. Changes in urine ion concentrations may be the major determinant of NKCC2A activity (load-dependent) whereas there is limited scope for variation in extracellular ion concentrations for NKCC1, and the actions of hormones and cytokines may be much more important. NKCC2A has a steadier work-load (in man 1 mole of Na^+^ is filtered each hour, day or night, and must be reabsorbed, dietary fluctuations represent a tiny fraction of this), whereas for NKCC1 activity may have to be low at rest but be capable of rising rapidly to deal with high peak demands. Strong activation by a fall, and inhibition by a rise in cell [Cl^−^] or [Na^+^] makes physiological sense for NKCC1, but not for NKCC2 where such effects would limit the ability of the transporter to respond to changes in urine ion concentration. Here, it is important that both Cl^−^ and Na^+^ are allowed to pass through the epithelium without raised cell levels unduly inhibiting further entry on the cotransporter.
